# Protein phosphatase PPM1A inhibition attenuates osteoarthritis via regulating TGF-**β**/Smad2 signaling in chondrocytes

**DOI:** 10.1172/jci.insight.166688

**Published:** 2023-02-08

**Authors:** Qinwen Ge, Zhenyu Shi, Kai-ao Zou, Jun Ying, Jiali Chen, Wenhua Yuan, Weidong Wang, Luwei Xiao, Xia Lin, Di Chen, Xin-Hua Feng, Ping-er Wang, Peijian Tong, Hongting Jin

**Affiliations:** 1 Institute of Orthopaedics and Traumatology, The First Affiliated Hospital of Zhejiang Chinese Medical University, Hangzhou, China.; 2 The First College of Clinical Medicine, Zhejiang Chinese Medical University, Hangzhou, China.; 3 Department of Orthopaedic Surgery, The First Affiliated Hospital of Zhejiang Chinese Medical University, Hangzhou, China.; 4 Department of Orthopedics, The Second Affiliated Hospital of Zhejiang Chinese Medical University, Hangzhou, China.; 5 Department of Hepatobiliary and Pancreatic Surgery and Zhejiang Provincial Key Laboratory of Pancreatic Disease, The First Affiliated Hospital, Zhejiang University School of Medicine, Hangzhou, China.; 6 Research Center for Human Tissues and Organs Degeneration, Shenzhen Institutes of Advanced Technology, Chinese Academy of Sciences, Shenzhen, China.; 7 The MOE Key Laboratory of Biosystems Homeostasis & Protection and Zhejiang Provincial Key Laboratory of Cancer Molecular Cell Biology, Life Sciences Institute and; 8 Life Sciences Institute, Zhejiang University, Hangzhou, China

**Keywords:** Bone Biology, Therapeutics, Cartilage, Osteoarthritis, Signal transduction

## Abstract

TGF-β signaling is crucial for modulating osteoarthritis (OA), and protein phosphatase magnesium–dependent 1A (PPM1A) has been reported as a phosphatase of SMAD2 and regulates TGF-β signaling, while the role of PPM1A in cartilage homeostasis and OA development remains largely unexplored. In this study, we found increased PPM1A expression in OA chondrocytes and confirmed the interaction between PPM1A and phospho-SMAD2 (p-SMAD2). Importantly, our data show that PPM1A KO substantially protected mice treated with destabilization of medial meniscus (DMM) surgery against cartilage degeneration and subchondral sclerosis. Additionally, PPM1A ablation reduced the cartilage catabolism and cell apoptosis after the DMM operation. Moreover, p-SMAD2 expression in chondrocytes from KO mice was higher than that in WT controls with DMM induction. However, intraarticular injection with SD-208, repressing TGF-β/SMAD2 signaling, dramatically abolished protective phenotypes in PPM1A-KO mice. Finally, a specific pharmacologic PPM1A inhibitor, Sanguinarine chloride (SC) or BC-21, was able to ameliorate OA severity in C57BL/6J mice. In summary, our study identified PPM1A as a pivotal regulator of cartilage homeostasis and demonstrated that PPM1A inhibition attenuates OA progression via regulating TGF-β/SMAD2 signaling in chondrocytes and provided PPM1A as a potential target for OA treatment.

## Introduction

Osteoarthritis (OA) is a common degenerative condition that affects more than 250 million people in varying degrees around the world (1). As a joint disease with high morbidity and disability, OA has caused considerable personal, economic, and societal burdens (2, 3). The pathological characteristic of OA is primarily associated with progressive cartilage destruction and is usually accompanied by subchondral bone sclerosis, synovial inflammation, and aberrant osteophyte formation (4, 5). Nonetheless, there is a lack of efficient disease-modifying intervention since the cellular and molecular mechanism of OA pathogenesis still remains to be elucidated.

It has been well established that TGF-β signaling plays an essential role in maintaining cartilage homeostasis as well as osteoarthritic development (6, 7). Accumulating evidence, both in vivo and in vitro, has emerged that ablation of TGF-β signaling components impairs chondrocyte homeostasis, increases production of matrix-degrading enzymes such as MMP-13 and a disintegrin and metalloproteinase with thrombospondin motifs-5 (Adamts-5), and eventually leads to cartilage damage or even spontaneous OA phenotypes (8–10). Otherwise, SMAD2/3 have been regarded as the pivotal signaling transducers in canonical TGF-β signaling pathway. The most critical step of canonical TGF-β pathway activation is the phosphorylation of SMAD2/3 induced by TGF-β receptor I kinase (TGFβRI) and then phospho-SMAD2/3 (p-SMAD2/3) combines with SMAD4 as a complex that shuttles into the nucleus to trigger further transcriptional responses (11, 12). Importantly, decreased phosphorylation level of SMAD2 in chondrocytes is known to be either a cause or a consequence of cartilage degeneration (13).

Protein phosphatase magnesium-dependent 1A (PPM1A) is a member of the metal-dependent protein phosphatases (PPMs) family that only dephosphorylate phospho-serine and phospho-threonine residues (14). Of note, PPM1A has been identified as a potent protein phosphatase of multiple substrates and SMAD2, the key regulatory factor in canonical TGF-β signaling, is one of them (15, 16). Considering that TGF-β signaling is an important pathway for cartilage homeostasis and PPM1A functions as an exclusive phosphatase for p-Smad2 and terminates TGF-β signaling, targeting PPM1A to modulate TGF-β signaling in chondrocytes during OA pathogenesis is an ambitious proposition.

In this study, to address our hypothesis, we have taken the initiative to determine the PPM1A expression in OA chondrocytes in vivo and in vitro and employed PPM1A KO mice to investigate the biological effect of PPM1A in a DMM-induced OA model in vivo. Here, we characterized that PPM1A-deficient mice following DMM surgery presented preserved articular cartilage and ameliorative OA development by regulating canonical TGF-β signaling in chondrocytes. We also confirmed the protective effect of administering Sanguinarine chloride (SC) or BC-21, as a PPM1A inhibitor, on DMM-induced OA progression, suggesting PPM1A could serve as a potential target for the management of OA.

## Results

### PPM1A expression was increased in human OA cartilage.

**Figure 1:**
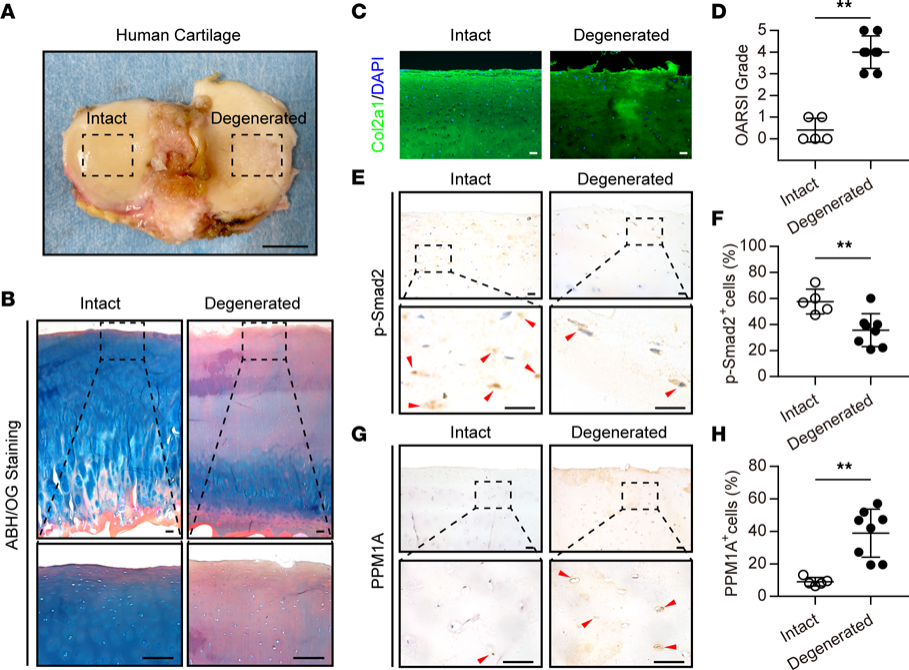
The expression of PPM1A is involved in human OA cartilage. (**A**) Human cartilage samples in the tibial plateau were obtained from patients with OA who received TKA surgery. Dashed boxes indicate representative areas of intact cartilage (*n* = 5 individuals) and degenerated cartilage (*n* = 8 individuals). Scale bar: 2 cm. (**B**) Representative images of ABH/OG staining for intact and degenerated cartilage. Scale bar: 200 μm. (**C**) Representative immunofluorescent staining for Col2a1 (green) of intact and degenerated cartilage, respectively, and DAPI (blue) was used to stain nuclei. Scale bar: 50 μm. (**D**) The degree of cartilage degradation is quantified according to the OARSI grade. (**E**) Representative IHC staining for p-SMAD2. Red arrowheads indicate positive cells. Scale bar: 25 μm. (**F**) Quantification of p-SMAD2 positive chondrocytes ratio in human cartilage. (**G**) Representative IHC staining for PPM1A. Red arrows indicate positive cells. Scale bar: 50 μm. (**H**) Quantification of PPM1A positive cells in human cartilage. Data were presented as means ± SD and **P < 0.01 by 2-tailed unpaired parametric Student’s *t* test or nonparametric Mann-Whitney *U* test.

To investigate the involvement of PPM1A in OA, we first analyzed the PPM1A expression in intact and degenerated human cartilage. In this study, human cartilage samples in tibial plateau were obtained from patients with OA who received total knee arthroplasty (TKA) surgery. Specifically, degenerated cartilage was mainly from the medial tibial plateau, which was coarse and jagged, while relatively intact cartilage was from the lateral area (Figure 1A). As expected, degenerated cartilage showed a substantial sulfated glycosaminoglycan (sGAG) depletion and almost calcified cartilage according to Alcian Blue Hematoxylin/Orange G (ABH/OG) staining (Figure 1B). Next, intact and degenerated cartilage samples were also authenticated by immunofluorescence for Col2a1, and data indicated a reduced Col2a1 expression in degenerated human cartilage (Figure 1C). Additionally, degenerated cartilage groups displayed a prominent increase on the Osteoarthritis Research Society International (OARSI) grade versus intact cartilage (Figure 1D). Consistent with prior findings (17, 18), here, we found that the phosphorylation level of SMAD2, a crucial regulator of canonical TGF-β signaling, in human degenerated cartilage was much lower than that in intact cartilage (Figure 1, E and F). Given that PPM1A functions as a dominating phosphatase of SMAD2, we next detected the PPM1A expression in arthritic chondrocytes. As expected, intact cartilage displayed a minimal PPM1A expression in chondrocytes. In contrast, PPM1A expression was dramatically increased in degenerated cartilage (Figure 1, G and H). Taken together, these data confirmed that TGF-β/SMAD2 signaling was downregulated in human OA cartilage and strongly indicated that PPM1A is perhaps an intrinsic negative regulator of TGF-β/SMAD2 signaling in articular chondrocytes.

### PPM1A expression negatively correlates with SMAD2 phosphorylation in chondrocytes during OA progression.

**Figure 2:**
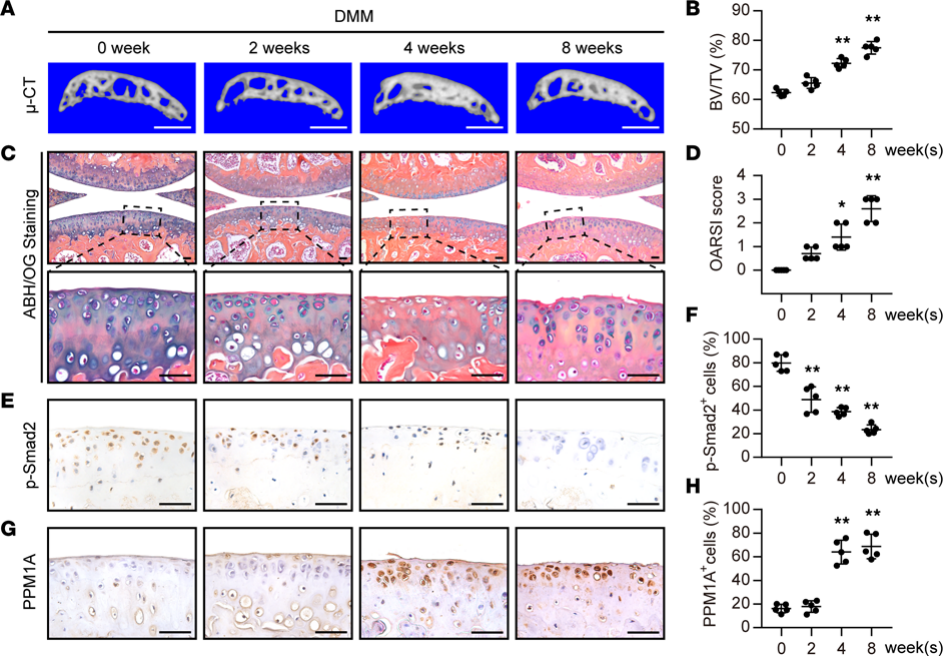
PPM1A expression is inversely related to SMAD2 phosphorylation in chondrocytes during OA progression. (**A**) Representative 3D reconstruction images of medial tibial subchondral bone after DMM surgery at different time points. Scale bar: 1 mm. (**B**) Quantification of BV/TV from medial tibial subchondral bone after DMM surgery. (**C**) ABH/OG staining for articular cartilage at different time points after DMM operation. Scale bar: 50 μm. (**D**) OARSI scores for the articular cartilage degeneration assessment at different time points. (**E**) Representative IHC staining for p-SMAD2 expression in articular cartilage. Scale bar: 50 μm. (**F**) Quantification of p-SMAD2 positive cells ratio in articular chondrocytes during OA progression. (**G**) Representative IHC staining for PPM1A expression in articular cartilage. Scale bar: 50 μm. (**H**) Quantification of p-SMAD2 positive cells ratio in articular chondrocytes during OA progression. All the data were presented as means ± SD, *n* = 5 mice per time point. *P < 0.05 and **P < 0.01 by 1-way ANOVA with Dunnett’s *t* test.

Next, we also investigated the canonical TGF-β signaling activity and PPM1A expression during the OA process in DMM-induced mice, a mechanical instability related OA model. The knee joints were harvested at different time points after DMM surgery. As the development of OA, we observed aggravating articular degeneration as well as subchondral bone deterioration in DMM mice. Specifically, according to the μ-CT data, 3D reconstruction of subchondral bone displayed an increased ratio of bone volume to tissue volume (BV/TV) at 4 weeks and 8 weeks after DMM surgery (Figure 2, A and B). Consistent with severe thickening of the subchondral bone, ABH/OG staining exhibited a decreased blue staining at the cartilage area during the OA course, indicating a progressive cartilage matrix loss at middle and late OA stages (Figure 2C). As expected, DMM surgery also increased OARSI scores of cartilage damage (Figure 2D). To understand the role of TGF-β signaling in articular chondrocytes in response to DMM induction, here, we examined the expression of p-SMAD2, which could indicate TGF-β signaling activity in chondrocytes. At the early OA stage, p-SMAD2 was abundantly expressed in chondrocytes while its expression was increasingly decreased with the progression of the disease (Figure 2, E and F). To evaluate whether the phosphorylation level of SMAD2 in chondrocytes is associated with PPM1A expression, we then carried out a time-course analysis of PPM1A expression in chondrocytes. IHC results revealed that PPM1A was almost undetectable in normal cartilage, was elevated after OA initiates, and maintained at a high level at 4 weeks and 8 weeks following DMM surgery (Figure 2, G and H). Otherwise, PPM1A in either subchondral bone or synovium tissue was lowly expressed and the DMM operation did not induce its expression in subchondral bone or synovium area significantly (Supplemental Figure 1; supplemental material available online with this article; https://doi.org/10.1172/jci.insight.166688DS1). Thus, these findings would seem to suggest that PPM1A expression was upregulated and it might negatively correlate with SMAD2 phosphorylation in chondrocytes in the process of OA.

### PPM1A interacted with p-SMAD2 in articular chondrocytes.

**Figure 3:**
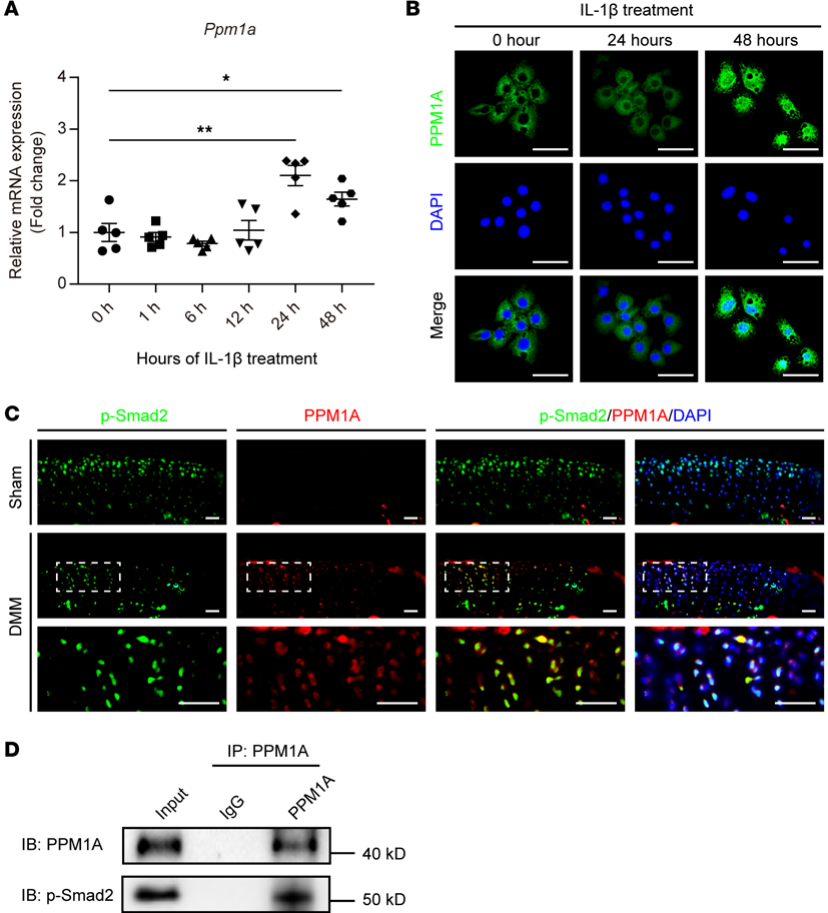
PPM1A interacted with p-SMAD2 in articular chondrocytes. (**A**) Fold changes of *PPM1A* mRNA expression in primary articular chondrocytes induced by IL-1β (10 ng/mL) treatment for indicated times. Data were presented as means ± SD and determined by 1-way ANOVA with Dunnett’s *t* test. *P < 0.05 and **P < 0.01. (**B**) Representative immunofluorescence images of PPM1A expression (green) in response to IL-1β (10 ng/mL) treatment for 24 hours or 48 hours. DAPI (blue) staining of nuclei. Scale bar: 50 μm. (**C**) Colocalization of PPM1A (red) and p-SMAD2 (green) in articular chondrocytes from sham and DMM mice at 4 weeks after surgery. Overlap between PPM1A and p-SMAD2 was presented as yellow. Nuclei were counterstained with DAPI (blue). Scale bar: 100 μm. (**D**) Co-IP assay for interactions between PPM1A and p-SMAD2 in primary articular chondrocytes treated with TGF-β1 (10 ng/mL) for 48 hours. *n* = 3.

Moreover, in vitro experiments showed that IL-1β treatment significantly increased the *PPM1A* gene expression in mouse primary articular chondrocytes (Figure 3A). The immunofluorescence results also revealed that IL-1β treatment induced PPM1A protein expression and promoted PPM1A entry into the nucleus (Figure 3B). In addition, we also performed colocalization of PPM1A and p-SMAD2 on articular cartilage in mice that received sham or DMM surgery. We found that the distribution of PPM1A expression at least partially overlapped with the p-SMAD2 expression in DMM mice (Figure 3C). We further carried out endogenous co-IP assay to determine whether PPM1A could bind with p-SMAD2 in chondrocytes. Of note, this result confirmed the physical interaction between PPM1A and p-SMAD2 in primary chondrocytes in vitro (Figure 3D). Accordingly, in combination with these in vitro and in vivo data, we proved the protein binding between PPM1A and p-SMAD2 in articular chondrocytes.

### PPM1A ablation attenuated OA progression associated with DMM mice.

**Figure 4:**
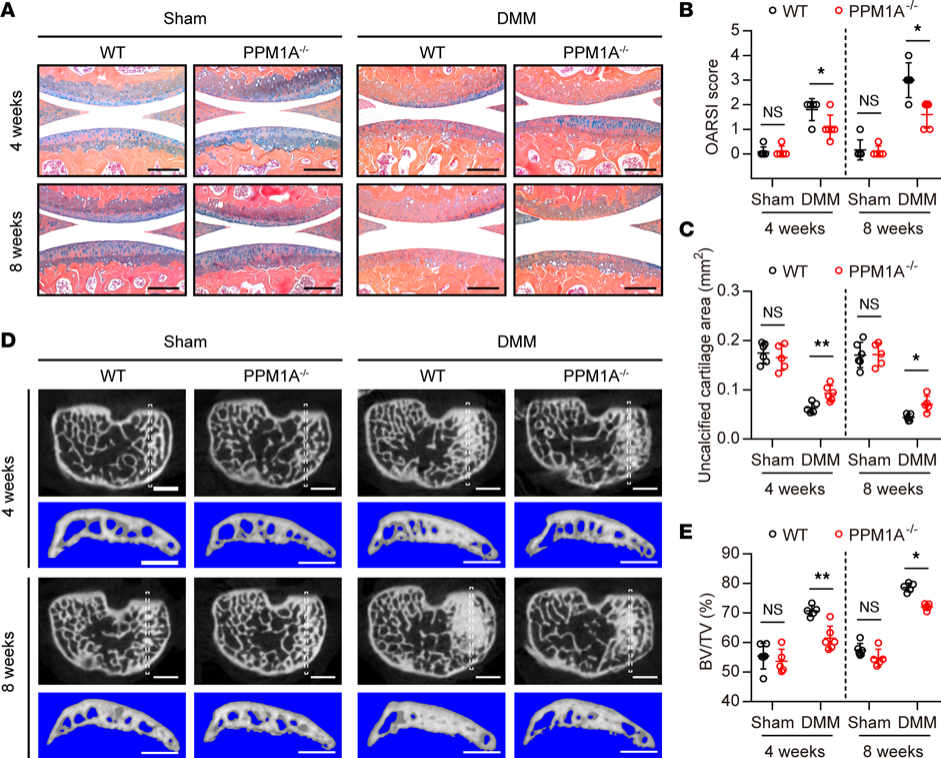
PPM1A deletion ameliorates cartilage degeneration and subchondral sclerosis in DMM-induced OA mice. (**A**) Representative images of ABH/OG staining for knee sections of WT and PPM1A-KO (PPM1A^−/−^) mice at 4 weeks and 8 weeks following DMM injury. Scale bar: 200 μm. (**B**) OARSI scores for assessment of the cartilage degeneration at 4 weeks and 8 weeks after surgery. (**C**) Histomorphometric quantification of uncalcified cartilage area in tibial and femoral articular cartilage per knee section. (**D**) Representative μ-CT images for transverse plane and 3D reconstruction of tibial subchondral bone compartment from WT and PPM1A^−/−^ mice at 4 weeks and 8 weeks after surgery. White dashed boxes in transverse plane images indicate the ROI for 3D reconstruction. Scale bar: 1 mm. (**E**) Quantification of tibial subchondral BV/TV at 4 weeks and 8 weeks after sham or DMM surgery. Data were presented as means ± SD and analyzed by 2-way ANOVA with Šidák post hoc analysis, *n* ≥ 5 mice per group. *P < 0.05, **P < 0.01.

Prior evidence has indicated that protein phosphatase PPM1A might play an important role in OA pathogenesis. Next, we examined whether PPM1A deletion affected the OA progression in DMM mice. For the testing of our hypothesis, we generated PPM1A global ablation mice and genotyping was confirmed by PCR (Supplemental Figure 2, A and B). PPM1A mutants were born at the expected Mendelian frequency as previously reported (19). Here, we found that there was no obvious difference in the gross appearance between the WT and PPM1A-KO newborn mice (Supplemental Figure 2C). Additionally, we also performed a whole skeleton staining on mice at P0 and the result showed that PPM1A ablation did not affect the embryonic development (Supplemental Figure 2, D and E) and postnatal development (Supplemental Figure 3, A and B). Furthermore, to evaluate whether PPM1A deficiency affects cartilage homeostasis, we performed histological morphological observations at both articular cartilage and growth plate cartilage stained with ABH/OG. Here, PPM1A^–/−^ mice presented a similar cartilage histomorphology as the WT littermates (Supplemental Figure 3C), and there was no significant difference in the thickness of articular cartilage and growth plate cartilage between the male WT and KO mice at 3 months old (Supplemental Figure 3, D and E). Similarly, sham-operated mice exhibited analogous cartilaginous phenotypes and with no significant difference in OARSI scores between the 2 strains. Of note, we found that PPM1A-KO mice that underwent DMM operation exhibited significantly less arthritic cartilage matrix degradation as determined by ABH/OG staining compared with WT mice at 4 and 8 weeks (Figure 4A). Furthermore, the average OARSI scores in the mutants was significantly lower than that in WT mice after DMM surgery (Figure 4B). Moreover, quantification of uncalcified cartilage area in knee sections also indicated that PPM1A ablation delayed cartilage calcification in DMM mice (Figure 4C). We next sought to gain further insight into other pathological changes of OA, such as subchondral bone sclerosis and synovitis. Results from μ-CT suggested that the subchondral bone microstructure was comparable between sham-operated WT and PPM1A-KO mice (Figure 4D). However, DMM induction caused a gradual sclerotization in the medial tibial subchondral bone in WT mice. In contrast, PPM1A^−/−^ mice presented reduced severity of subchondral thickening at 4 and 8 weeks following DMM surgery as demonstrated by transverse plane images of the subchondral bone plate and 3D reconstruction images of subchondral bone (Figure 4D). Quantitative data confirmed that BV/TV of medial tibial subchondral bone was decreased in KO mice compared with WT at the corresponding time point (Figure 4E). Similarly, 3D reconstruction images of general knee joint showed that PPM1A^−/−^ mice that received DMM induction had less osteophyte formation compared with WT mice, as measured by the osteophyte score (Supplemental Figure 4, A–C). In contrast, PPM1A ablation had little protection against synovitis induced by DMM operation (Supplemental Figure 4, D and E). Therefore, these consistent observations provided in vivo evidence that PPM1A genetic inhibition could alleviate cartilage degeneration as well as subchondral bone sclerosis in injury-induced OA.

### PPM1A ablation prevented cartilage catabolism and SMAD2 dephosphorylation in DMM-induced mice.

**Figure 5:**
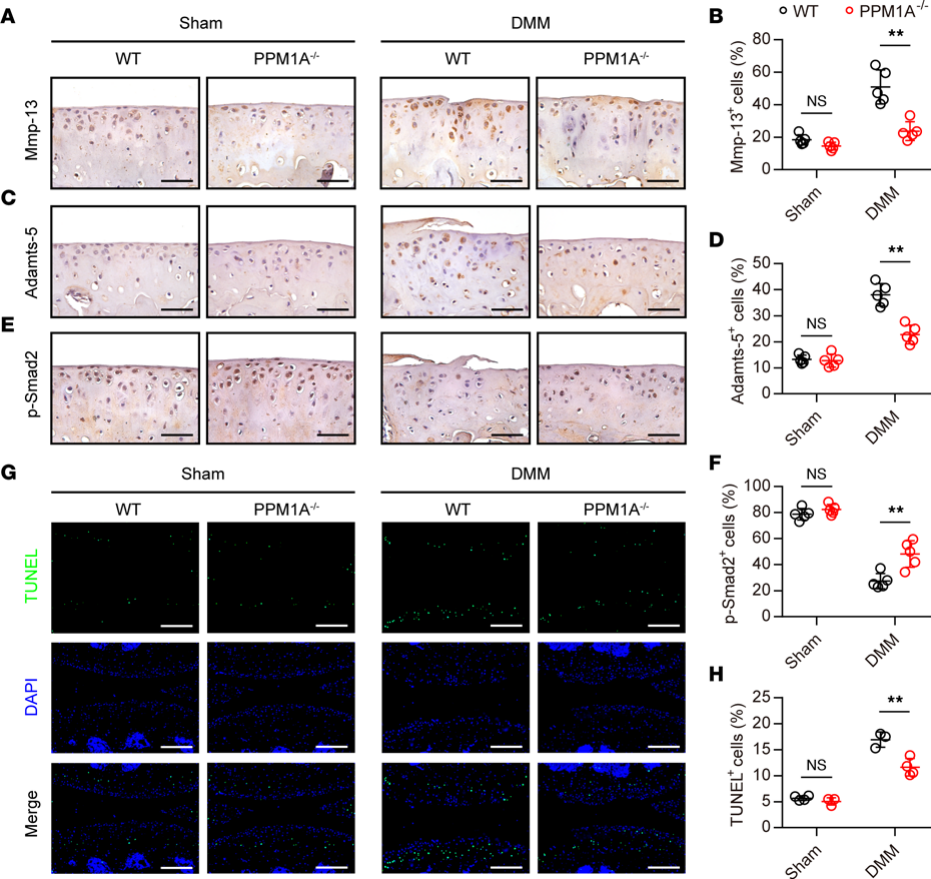
PPM1A ablation prevented cartilage catabolism and SMAD2 dephosphorylation in chondrocytes responding to DMM surgery. (**A**) IHC expression of MMP-13 in knee joints from WT mice and PPM1A^−/−^ mice at 8 weeks after DMM surgery. Scale bar: 50 μm. (**B**) Quantification for MMP-13 expression in chondrocytes from WT and KO mice at 8 weeks following operation. (**C**) Representative images of Adamts-5 IHC expression from WT mice and PPM1A^−/−^ mice at 8 weeks after DMM surgery. Scale bar: 50 μm. (**D**) IHC quantification for Adamts-5 expression in chondrocytes from mice at 8 weeks. (**E**) Representative images of p-SMAD2 expression in articular chondrocytes from WT mice and PPM1A^−/−^ mice at 8 weeks after DMM surgery. Scale bar: 50 μm. (**F**) IHC quantification for p-SMAD2 expression in chondrocytes from mice at 8 weeks following sham or DMM surgery. (**G**) TUNEL staining for apoptosis chondrocytes in articular cartilage from WT mice and PPM1A^−/−^ mice at 8 weeks after operation. Scale bar: 200 μm. (**H**) Quantification for TUNEL positive cells in articular cartilage. Data were presented as means ± SD and statistical analyses were performed by 2-way ANOVA with Šidák post hoc test, *n* ≥ 3 mice per group. **P < 0.01.

Since PPM1A-KO mice presented a distinct protection against DMM-induced OA phenotype, we then performed IHC analyses to further identify the role of PPM1A in cartilage catabolism following DMM surgery. Immunostaining for MMP-13, a key factor for cartilage matrix degradation, revealed a handful of MMP-13 positive chondrocytes in sham-operated mice of 2 strains. With DMM induction, the expression of MMP-13 was dramatically elevated in WT mice but this enhancement was slowed down with PPM1A deletion (Figure 5, A and B). Similarly, compared with WT mice, PPM1A-KO mice had a reduced expression of Adamts-5 in chondrocytes, suggesting less loss of aggrecan in response to DMM operation (Figure 5, C and D). Since our prior study fully demonstrated that MMP-13 and Adamts-5 were 2 critical downstream targets of TGF-β signaling (10), immunostaining for p-SMAD2 was subsequently performed to determine the role of PPM1A in regulating canonical TGF-β signaling during OA pathogenesis. As expected, the phosphorylation level of SMAD2 in chondrocytes from PPM1A^−/−^ mice maintained a relatively higher level versus WT mice with DMM induction. In other words, PPM1A deficiency, at least in part, reduced the dephosphorylation of p-SMAD2 during OA progression (Figure 5, E and F). Here, in addition, we carried out TUNEL assay to examine whether PPM1A ablation could protect chondrocytes against cell apoptosis in DMM mice. Immunofluorescence staining showed decreased apoptosis in DMM-induced chondrocytes in PPM1A^−/−^ mice compared with that in WT mice (Figure 5, G and H). Thus, these results indicated that PPM1A deficiency significantly reduced the expression of cartilage-degrading enzymes and protected against chondrocyte apoptosis possibly by modulating the TGF-β/SMAD2 signaling activity.

### TGF-β/SMAD2 inhibition abolished the OA-protective phenotypes in PPM1A-KO mice.

**Figure 6:**
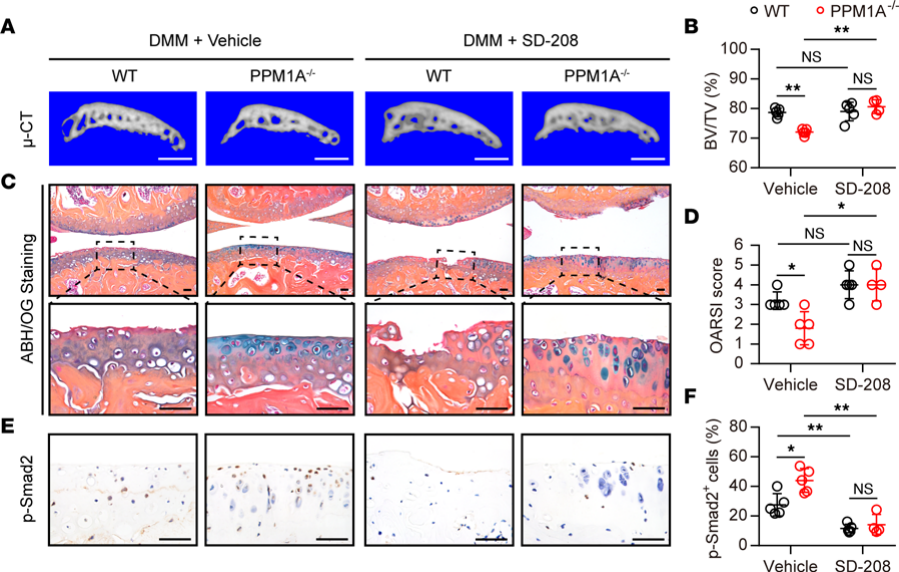
Treatment with SD-208 abolished the protective effect of PPM1A^−/−^ mice on articular cartilage after DMM surgery. (**A**) Representative 3D reconstructed images of subchondral bone responding to DMM surgery at 8 weeks. Scale bar: 1 mm. (**B**) Quantitative data of subchondral BV/TV from WT mice and PPM1A^−/−^ mice at 8 weeks after DMM surgery. (**C**) Representative images of ABH/OG staining for knee sections from WT mice and PPM1A^−/−^ mice treated with SD-208 for 8 weeks. Scale bar: 200 μm. (**D**) Quantitative data of OARSI scores from WT mice and PPM1A^−/−^ mice at 8 weeks after DMM surgery. (**E**) Representative images of IHC analyses for p-SMAD2 from WT mice and PPM1A^−/−^ mice treated with SD-208 for 8 weeks after DMM operation. Scale bar: 50 μm. (**F**) Quantification for p-SMAD2 positive chondrocytes ratio from mice at 8 weeks after surgery. Data were presented as means ± SD and statistical analyses were performed by 2-way ANOVA with Šidák post hoc test, *n* ≥ 4 mice per group. *P < 0.05, **P < 0.01.

Based on the above evidence, we anticipated that the loss of PPM1A reduced the expression of cartilage-degrading enzymes and ameliorated cartilage degeneration via canonical TGF-β signaling. To verify this hypothesis, we employed SD-208, a selective inhibitor for TGFβRI, to block the canonical TGF-β/SMAD2 signaling (20, 21). In the present study, SD-208 were administrated intraarticularly twice a week for 8 weeks since DMM initiation. The 3D-construction for subchondral bone showed that the subchondral sclerosis suppressing effect of PPM1A-KO mice was revoked by SD-208 treatment (Figure 6, A and B). In addition, PPM1A-KO mice still displayed an obviously protective effect on cartilage degeneration with vehicle treatment when compared with the WT group (Figure 6C). In contrast, with SD-208 administration, both WT and KO mice exhibited severe cartilage damage as determined by ABH/OG staining (Figure 6C) and the OARSI score showed that there was no statistical difference between the 2 strains (Figure 6D). Notably, intraarticular injections of SD-208 caused a potent inhibition for canonical TGF-β signaling activity as confirmed by an apparent decrease of p-SMAD2 expression in arthritic chondrocytes from PPM1A^−/−^ mice (Figure 6, E and F). Here, we provided further in vivo evidence that PPM1A blockade alleviated OA process, at least in part, through regulating the TGF-β/SMAD2 signaling pathway.

### Pharmacologic PPM1A inhibition alleviated OA severity in WT mice.

**Figure 7:**
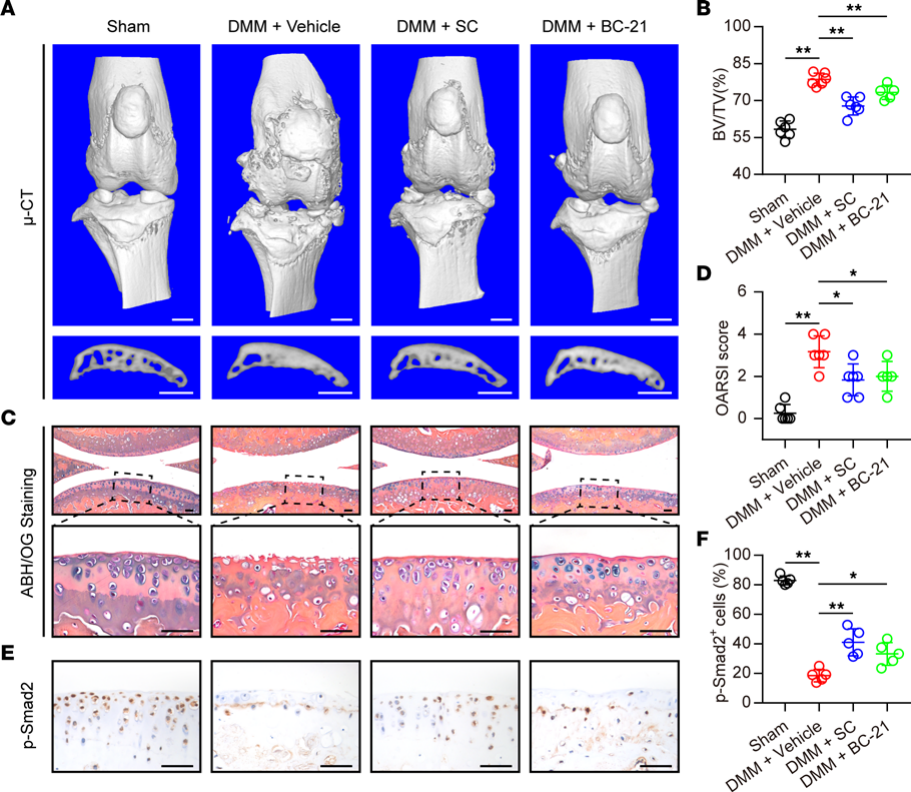
Intraarticular injection of PPM1A inhibitor alleviated OA severity in DMM mice. (**A**) Representative images of 3D reconstruction for medial tibial subchondral bone at 8 weeks after sham or DMM surgery. Mice were treated with vehicle, SC, or BC-21 every 5 days after DMM surgery. All the mice were sacrificed at 8 weeks after surgery. Scale bar: 1 mm. (**B**) Quantitative data of BV/TV in subchondral bone from WT and KO mice at 8 weeks after sham or DMM surgery. (**C**) Representative images of ABH/OG-stained sections of knee joints. Scale bar: 50 μm. (**D**) Quantitative data of OARSI score from WT mice and PPM1A^−/−^ mice at 8 weeks after surgery. (**E**) Immunostaining of p-SMAD2 in articular cartilage at 8 weeks after sham or DMM surgery. Scale bar: 50 μm. (**F**) Quantification of p-SMAD2 positive chondrocytes. Data were presented as means ± SD and *n* ≥ 5 mice per group. *P < 0.05, **P < 0.01 by 1-way ANOVA with Dunnett’s *t* test.

To better characterize the protective role of PPM1A blockade during OA development, we employed SC and BC-21, which have been considered as small-molecule PPM1A inhibitors (16, 22). Here, these 2 inhibitors were administrated by intraarticular injection to determine the effect on articular chondrocytes in response to OA induction. Results from μ-CT analyses displayed that DMM surgery induced substantive osteophyte formation as well as severe subchondral sclerosis while treatment with PPM1A inhibitor blunted these OA pathological changes (Figure 7, A and B). Additionally, dramatic cartilage lesion was observed in ABH/OG staining in the DMM group, which was treated with vehicle only. Importantly, intraarticular injection of SC or BC-21 both preserved integrity of the articular cartilage and protected against injury-induced degeneration (Figure 7C), and OARSI scores in the inhibitor group were lower than those in the vehicle group (Figure 7D). Nevertheless, both 2 small-molecule PPM1A inhibitors failed to mitigate DMM-induced synovitis and there was no significant difference in the synovitis score between the vehicle group and the inhibitor group (Supplemental Figure 5), which was consist with genetic inhibition in PPM1A-KO mice. Next, we evaluated the regulations of these inhibitors on the canonical TGF-β signaling pathway in chondrocytes during OA progression. By IHC staining for p-SMAD2, we found that DMM operation markedly decreased the phosphorylation levels of SMAD2 in chondrocytes, which was largely reversed by intraarticular injection of SC or BC-21 (Figure 7, E and F). Collectively, these results confirmed pharmacologic inhibition of PPM1A could slow down OA development, suggesting PPM1A was a potential target for OA therapy.

## Discussion

TGF-β signaling plays an essential role in cartilage homeostasis and OA progression. The initiating step in TGF-β signaling cascades is that the TGF-β ligand binds to the TGF-β receptor type II (TGFβRII), which then recruits TGFβRI to trigger a signal transduction cascade. Our previous study has determined the chondrocyte-specific deletion of TGFβRII, the upstream component of TGF-β signaling, upregulates MMP-13 and Adamts-5 expressions, increases cartilage catabolism, and leads to an OA-like phenotype in mice, suggesting inactivation of TGF-β signaling is involved in OA initiation (10). Furthermore, in canonical TGF-β signaling, SMAD2, as well as SMAD3, is regarded as receptor SMAD (R-SMAD), whose phosphorylation is mediated by the receptor kinase TGFβRI (23). It has been illustrated that whether in spontaneous OA model in STR/ort mice or the instability-associated OA model treated with collagenase, the TGF-β expression and SMAD2 phosphorylation level in articular cartilage is gradually reduced as the OA disease progresses (24), which is consistent with our results in human OA cartilage and mice DMM-treated articular chondrocytes. Accordingly, these findings suggests that TGF-β/SMAD2 is highly associated with cartilage homeostasis and has been recognized as a pivotal factor in OA pathogenesis. Even so, the precise regulation of p-SMAD2 levels, especially the dephosphorylation, in chondrocytes remains largely unexplored.

Reversible protein phosphorylation that is mainly regulated by kinases and phosphatases is a fundamental and critical modification in numerous signaling transductions and biological processes (25). In canonical TGF-β signaling, the C-terminal SXS motif of p-SMAD2 and p-SMAD3 are dephosphorylated by protein phosphatase to regulate the signaling activity (12). PPM1A, similar to many other phosphatases, is able to regulate multiple cellular functions, such as differentiation, proliferation, and immunity, that have been intensively investigated in cancer and metabolic diseases (26–29). Previous publications have reported that PPM1A modulates TGF-β signaling by dephosphorylating and inactivating SMAD2 in epithelial cell, keratinocyte, and bladder cancer cells (15, 30, 31). Hence, we proposed that PPM1A is likely to be an essential regulator of the TGF-β/SMAD2 signaling pathway whose dysregulation disrupts chondrocyte homeostasis and governs the pathogenesis of OA. Until now, to our knowledge, little work has been performed on the role of PPM1A in chondrocyte and OA conditions.

In the current study, we first identified that PPM1A expression was distinctly increased in chondrocytes in vivo and in vitro, which contributes to the pathogenesis of OA. Of note, its expression was negatively correlated with the SMAD2 phosphorylation level in chondrocytes, indicating that PPM1A may function as phosphatase to regulate p-SMAD2 expression by enhancing dephosphorylation of SMAD2. Though it has confirmed that PPM1A could directly combine with p-SMAD2 in epithelial cell (15), this biological interaction in chondrocytes still remains enigmatic. Given that, we carried out co-IP in vitro and colocalization in vivo to establish the protein interaction between PPM1A and p-SMAD2 in articular chondrocytes. Additionally, we noted that early loss of p-SMAD2 at 2 weeks after DMM surgery is driven independently of PPM1A. Recently, Li et al. (18) have demonstrated that the expression of TGFβRII receptor in articular cartilage decreased as early as 3 days after DMM surgery in mice, which may result in reduced combination and activation of TGFβRII and TGFβRI and further cause low activity as a kinase for p-SMAD2. Additionally, though the protein phosphorylation level of SMAD2 is mainly controlled by the kinases and phosphatases, it could also be directly degraded by ubiquitination and proteolysis under certain conditions (32, 33). Hence, the decreased phosphorylation level of SMAD2 in the early stage might be mainly explained by the decreased kinase activity or increased degradation.

To further explore the biological role of PPM1A in vivo, we generated PPM1A-KO mice. In line with previous investigations (19, 30), our data showed that PPM1A^−/−^ mice developed without any gross morphological abnormality from neonate to adulthood. However, a recent study showed that macrophage-specific deletion of PPM1A expression with LysM-Cre resulted in PPM1A^fl/fl^ mice having a slightly smaller body type when compared with LysM-Cre control mice at 6 weeks of age (34). We suspect that loss of PPM1A in other cell populations may compensate for body size phenotype cause by PPM1A deficiency in macrophages. Meanwhile, there was no significant difference in cartilage development, including growth plate cartilage and articular cartilage, between male PPM1A-KO mice and WT controls. Nonetheless, we have also noted that the IMPC website (https://www.mousephenotype.org/data/genes/MGI:99878) reported that female PPM1A^–/–^ mice exhibit significant phenotypes in decreased bone mineral content, abnormal bone structure, short tibia, and increased grip strength, indicating that the PPM1A mediation of skeletal development might be in a sex-dependent manner. To our knowledge, these results, for the first time, indicated that PPM1A is likely to be dispensable for limb bud development and cartilage development in male mice.

Importantly, in this study we found that PPM1A ablation repressed cartilage catabolism, reduced chondrocyte apoptosis, and attenuated cartilage degeneration in DMM mice. Our previous research has clarified that KO of *MMP13* or *Adamts5* gene in chondrocytes could significantly alleviate the OA-like phenotypes caused by the loss of TGF-β signaling, which concluded that both MMP-13 and Adamts-5 are critical downstream targets involved in TGF-β signaling pathways during cartilage homeostasis (10, 35). In contrast, upregulation of TGF-β signaling in chondrocytes is able to suppress the production of MMP-13 and Adamts-5, which also functions as matrix-degrading enzymes (36, 37). According to our data, with OA induction, the rates of chondrocytes positive for MMP-13 and Adamts-5 in KO mice were significantly lower than those in WT mice. In the meantime, the p-SMAD2 expression in chondrocytes from PPM1A^−/−^ mice was higher than that in WT mice in response to DMM operation. Consequently, we reasoned that loss of PPM1A most likely maintained low levels of catabolism in DMM-induced chondrocytes by reducing the dephosphorylation of SMAD2.

It is worth noting that PPM1A is able to catalyze the dephosphorylation of multiple substrates besides p-SMAD2. For instance, PPM1A is able to dephosphorylate and inactivate p38 in the stress-responsive MAPK cascades (38). Also, previous research has reported that PPM1A acts as IKK-β phosphatases to terminate NF-κB activation, which was induced by TNF-α in HEK 293 T cells (39). More recently, PPM1A is also thought to be a phosphatase that dephosphorylates and activates the Yes-associated protein (YAP) to modulate mammalian intestinal and liver regeneration (40). To explore the potential mechanism, we treated PPM1A-KO mice with SD-208 to block the TGF-β/SMAD2 signaling and further clarified whether the chondroprotective action of PPM1A inhibition is mainly through TGF-β signaling or some other pathway. As expected, intraarticular injection with SD-208, a potent and specific inhibitor for TGFβRI, significantly repressed the phosphorylation of SMAD2 and blocked the TGF-β signaling in articular chondrocytes, which was in accordance with previous research results (20, 21). Notably, our results illustrated TGF-β/SMAD2 signaling inhibition revoked the OA-protective phenotypes in PPM1A-KO mice, suggesting PPM1A inhibition is a TGF-β signaling dependent manner in OA conditions. However, since TGFβRI may phosphorylate other substrates like pSMAD3 or even other noncanonical TGF-β signaling targets and SD-208 treatment may influence other pathways, here, we are still unable to completely exclude a potential contribution of other pathways to the observed effects.

During the past decade, it has been appreciated that the subchondral bone microenvironment is an emerging and crucial regulator in OA pathogenesis (41). Furthermore, the primary site for OA pathogenesis, articular cartilage or subchondral bone, is still a controversial issue (42). It has been proposed that aberrant activation of TGF-β in subchondral bone would contribute to articular cartilage degradation in OA and rheumatoid arthritis (RA) progression (43–45). Furthermore, deletion of the *Tgfbr2* gene in *Nestin*-positive mesenchymal stromal cells in subchondral bone or local subchondral administration of TGF-β-neutralizing Ab 1D11 could attenuate anterior cruciate ligament transection–induced (ACLT-induced) OA development (43). These findings may imply a biphasic regulatory role of TGF-β signaling in articular cartilage and subchondral bone. Therefore, to mainly repress the TGF-β signaling activity in articular chondrocytes and avoid directly affecting the subchondral bone area, we utilized SD-208 locally by intraarticular injection rather than i.p. injection in this study.

Although it has been confirmed that PPM1A is associated with osteogenesis and osteoclast commitment (34, 46, 47), our data showed that PPM1A deletion displayed comparable subchondral bone structure like WT controls before DMM induction. However, we found little expression of PPM1A in subchondral bone in physiological condition and no obvious alteration of PPM1A expression in the subchondral bone area after DMM surgery. Moreover, PPM1A ablation dramatically ameliorated subchondral sclerosis in DMM mice. On the basis of these findings, we supposed that the observed OA phenotypes in subchondral bone in KO mice are probably secondary to articular cartilage alteration rather than the direct regulation from loss of PPM1A in the cell population involved in subchondral bone. As for synovial inflammation, commonly considered a secondary pathological feature in response to DMM injury (48), our data showed that PPM1A in synovium tissue of knee joints was lowly expressed and was not significantly induced by DMM operation in mice, which may explain, at least in part, that PPM1A inhibition had no apparent protection against synovitis in the DMM model. In agreement with our results, IHC analysis of human synovial tissue specimens revealed that the PPM1A protein expression was much lower in synovial tissue derived from patients with OA than that in people with RA or ankylosing spondylitis (AS) (46), indicating a dispensable role of PPM1A for synovial inflammation in OA.

Although the precise mechanism by which PPM1A expression is increased in response to OA initiation is not fully elucidated in this study, our in vitro results revealed that an inflammatory factor, such as IL-1β, is perhaps a potent inducer of PPM1A production in chondrocytes. Similarly, it has been implicated that TNF stimulation induced PPM1A expression while pretreatment with NF-κB inhibitors could abolish this increased expression in human chronic myelogenous K562 leukemia cells, indicating PPM1A is likely to be a downstream target of the TNF/NF-κB signaling pathway (49). Moreover, a recent study revealed that extracellular PPM1A induces TNF production in macrophages via TLR4 and myeloid differentiation factor 88 (MyD88) pathway, and PPM1A levels in RA synovial fluid were positively correlated with inflammation (50). These results indicate that PPM1A expression and inflammation may interact and reinforce each other in OA disease.

This study still has several limitations. First, since we employed PPM1A global ablation mice rather than chondrocyte-specific conditional KO mice, our results cannot completely rule out the possibility that the observed OA phenotypes regulated by the loss of PPM1A in some other tissue went undetected in the present study. Second, we did not evaluate the effect of PPM1A ablation on OA-related pains such as gait behavior and von Frey behavioral pain. Third, we still did not completely exclude the possibility that SC as well as BC-21 may be involved in the regulation of other targets. In addition, the upstream regulatory mechanism of PPM1A expression in OA pathogenesis is worth further study.

In conclusion, to our knowledge, we characterized for the first time that PPM1A expression is upregulated in OA chondrocytes and PPM1A inhibition attenuates OA progression through regulating canonical TGF-β signaling activity. These findings provide new insight into canonical TGF-β signaling mechanisms responsible for OA disease and suggest PPM1A as a potentially novel therapeutic target for OA treatment.

## Methods

### Human cartilage samples.

In the current study, human cartilage samples were obtained from patients with OA who underwent TKA in the Department of Orthopedic Surgery at The First Affiliated Hospital of Zhejiang Chinese Medical University. All the procedures were approved by the Ethics Committee of The First Affiliated Hospital of Zhejiang Chinese Medical University (2019-KL-041-01). Human samples were fixed in 4% paraformaldehyde (PFA), decalcified in 14% EDTA, and paraffin embedded for further histological ABH/OG staining. Finally, the degree of cartilage damage was determined by gross and histopathological observation according to the OARSI Osteoarthritis Cartilage Histopathology Assessment System.

### Mice.

For all experiments, to rule out gender differences, only male C57BL/6J (WT) mice and male PPM1A-KO mice with a C57BL/6J background were employed and analyzed. We purchased male C57BL/6J mice from Zhejiang Chinese Medical University Laboratory Animal Research Center and PPM1A-KO mice were generated by a homogenous recombination strategy, in which the gene-trap vector was inserted in the intron 1 of PPM1A genomic locus. Primer sequences for *PPM1A* genotyping were presented in Supplemental Table 1. All the animal operating procedures in this study were approved by the Experimental Animal Ethics Committee of Zhejiang Chinese Medical University (202006-0244).

### DMM surgery.

Based on prior research (51), in the current study, DMM surgery was employed for mice OA induction due to its great ease and reproducibility. In brief, 10-week-old mice were anesthetized i.p. with 0.3% pentobarbital sodium. A capsulotomy was then performed**,** and the medial meniscotibial ligament (MMTL) from the right knee joint was dissected completely to cause instability of the medial meniscus. In the sham-operated group, mice were anesthetized and subjected to capsulotomy without MMTL dissection. Mice were sacrificed after 0, 2, 4, and 8 weeks, respectively, and right knee joints were collected for further experiments.

### Drug treatments.

In the present study, to fully address whether canonical TGF-β/SMAD2 signaling mediated the action of PPM1A on cartilage during OA development in vivo, both 10-week-old male WT and PPM1A-KO mice were treated with SD-208 (Selleck, CAS 627536-09-8; 20 μM), a specific inhibitor of TGFβRI, by intraarticular injection with a volume of 10 μL twice a week for 8 weeks after DMM operations. In addition, for the PPM1A-inhibitor treatment study, 10-week-old male C57BL/6J mice that received DMM operation were intraarticularly injected with PPM1A inhibitors, SC (MUST, CAS 5578-73-4; 5 μM) and BC-21, also known as NSC109268 (Millipore Sigma, CAS 691005-38-6; 100 μM), respectively. Specifically, 10 μL of inhibitor or PBS as vehicle was injected into the right knee joint cavity every 5 days for 8 weeks.

### μ-CT analysis.

All the samples were scanned by a high-resolution μ-CT scanner (SkyScan, 1176) and the scanning parameters were set to 45 kV, 500 μA, and a 780 ms exposure time. The region of interest (ROI) for evaluation was drawn from subchondral bone of the medial tibial plateau compartment and 5 consecutive sagittal plane images of ROI were used for 3D reconstruction by visualization software (SkyScan, CTVolx, v3.0). In addition, the quantitation of BV/TV of the ROI were collected from analysis software (SkyScan, CTAn, v1.15).

### Histology.

For histological investigation, knee samples were dipped in 4% PFA for 3 days and then decalcified in 14% EDTA solution for 14 days at room temperature. Subsequently, samples were dehydrated by an automatic dehydrator (Tissue-Tek VIP 5Jr) following paraffin embedding. Paraffin blocks were sectioned sagittally into 3 μm thick slices for staining. Following deparaffinization and rehydration, sections were stained with ABH/OG for morphologic observation. Here, we adopted the OARSI score to appraise the degree of articular cartilage degeneration, while osteophyte scores and the synovitis score described in previous studies were employed for assessments (52, 53). Uncalcified cartilage area of knee joint sections was quantificated by ImageJ software (NIH).

### IHC and immunofluorescence.

For IHC, sections were toasted at 60°C for 4 hours before deparaffinization and dehydration. Slices were then soaked in sodium citrate solution and heated at 60°C for 4 hours to make antigen retrieval. Subsequently, sections were incubated with primary Abs at 4°C overnight and treated for secondary Abs for 20 minutes the next day. The positive signal was visualized with DAB reagents (ZSGB-BIO) and slices were counterstained with hematoxylin. For immunofluorescence (IF), samples were incubated with a fluorescent secondary Ab for 40 minutes at room temperature and DAPI was used for nuclear staining. The primary Abs employed in the current study include Col2a1 (Abcam, catalog ab34712; 1:500, IF), PPM1A (Bioss, catalog bs-4162R; 1:300, IHC; and Abcam, catalog ab14824; 1:100, IF), MMP-13 (Abcam, catalog ab39012; 1:300, IHC), Adamts-5 (Bioss, catalog bs-3573R; 1:200, IHC), and p-SMAD2 (Ser465 and Ser467) (Thermo Fisher Scientific, catalog 44-244G; 1:300, IHC/IF). Quantitative analysis was performed using ImageJ software.

### TUNEL assay.

To evaluate the apoptotic cells in the articular cartilage, we performed a TUNEL assay according to the manufacturer’s guideline (Beyotime, catalog C1088). Briefly, following deparaffinage and rehydration, sections were permeabilized with DNase-free Proteinase K (20 μg/mL) for 15 minutes at 37°C. Subsequently, slides were treated with TUNEL solution and incubated at 37°C for 1 hour in a dark environment and counterstained with DAPI for 5 minutes. Finally, TUNEL positive cells were detected by fluorescence microscope.

### Whole skeleton staining.

Whole-mount skeletal staining was conducted to investigate the skeleton composition at P0. In brief, specimens were placed in acetone to permeabilize cell membranes and permit penetration of the stain following fixation in 95% ethyl alcohol overnight at room temperature. Then, specimens were incubated in Alcian blue and Alizarin red solution for cartilage and bone staining, respectively. Ultimately, samples were transferred in 100% glycerol for long-term storage. Analysis of the skeletal phenotype between WT and PPM1A-KO mice was performed with stereo microscopes.

### Cell culture.

For isolation of primary articular chondrocytes, cartilaginous femoral caps were removed from the femoral head of WT mice sacrificed at 2–3 weeks of age. Concretely, mice were sacrificed and disinfected in 75% ethyl alcohol prior to sampling. Then the femoral cartilaginous caps from bilateral femoral heads were digested by DMEM Nutrient Mixture F-12 (DMEM/F-12) medium (Thermo Fisher Scientific) containing 3 mg/mL Collagenase-D (Roche) and 1% penicillin/streptomycin (Thermo Fisher Scientific) for 6 hours at 37°C with intermittent percussion. Following digestion, the chondrocytes were harvested and cultured in complete DMEM/F-12 medium with 10% FBS. In this study, primary chondrocytes were not passaged and treatments were completed within 7 days of isolation from neonatal mice. For experiments in vitro, primary chondrocytes were exposed to recombinant mouse IL-1β (Sigma) at 10 ng/mL.

### Real-time quantitative PCR.

The total RNA was harvested and extracted from chondrocyte culture using TRIzol reagent (Sigma). TaqMan Reverse Transcription kit (Biomake) was used to reverse transcribe mRNA into complementary DNA. After this, the real time quantitative PCR (RT-qPCR) was conducted with SYBR Premix Ex Taq II (Takara). The primer sequences for the target genes used in this study were listed in Supplemental Table 2. The results of this experiment will provide the basis for β *actin* used as a reference gene to analyze quantitatively.

### Co-IP.

Endogenous co-IP was carried out to analyze the interaction between PPM1A and p-SMAD2 using primary articular chondrocytes from WT mice. Cells were lysed with Cell Lysis Buffer (Beyotime, catalog P0013) at 4°C for 30 minutes and centrifuged at 13,400*g* for 10 minutes, the supernatant was incubated with control IgG Ab (ABclonal, catalog AC005; 1:50) or anti-PPM1A Ab (Bioss, catalog bs-4162R; 1:50) and rotated overnight at 4°C. After that, the supernatants were added with Protein A+G Agarose beads (Beyotime, catalog P2055) and rotated at 4°C for another 3 hours. Then, the samples were centrifuged at 600*g* for 5 minutes and were subjected to SDS-PAGE and Western blotting. The Abs employed for Western blot containing PPM1A (Abcam, catalog ab14824; 1:1,000) and p-SMAD2 (Ser465, Ser467) (Thermo Fisher Scientific, catalog 44-244G; 1:1,000).

### Statistics.

All the data in the present study were presented as mean ± SD and the statistical analyses were performed with software GraphPad Prism (version 7.0). For comparison between 2 groups, 2-tailed unpaired parametric Student’s *t* test was used, and statistical analyses for comparison of more than 2 groups were performed by 1-way ANOVA followed by Dunnett’s *t* test or 2-way ANOVA followed by Šidák post hoc test. Data based on grading systems were analyzed using nonparametric Mann-Whitney *U* test (for comparisons of 2 samples) or Kruskal-Wallis test (for comparisons of multiple groups). For in vitro cell culture experiments, observations were repeated independently at least 3 times, and only data from a representative experiment are presented. Values of *P* < 0.05 were regarded as statistically significant.

### Study approval.

The human cartilage samples in the current study were obtained from patients with OA who underwent TKA in the Department of Orthopedic Surgery at The First Affiliated Hospital of Zhejiang Chinese Medical University and all the procedures were approved by the Ethics Committee of The First Affiliated Hospital of Zhejiang Chinese Medical University (2019-KL-041-01). All the animal operating procedures were approved by the Experimental Animal Ethics Committee of Zhejiang Chinese Medical University (202006-0244).

## Author contributions

QG, XHF, PW, PT, and HJ conceptualized the study. QG conducted the in vivo experiments. ZS and KZ collected the human samples and performed the in vitro assay. JY, JC, WY, and WW analyzed the data. QG wrote the original draft and LX, XL, DC, XHF, PW, PT, and HJ revised the manuscript critically for important intellectual content. PT and HJ supervised this study. All the authors read and approved the final version of this manuscript.

## Supplementary Material

Supplemental data

## Version 1 02/08/2023

Electronic publication

## Version 2 02/14/2023

Revision to affiliation 5
