# Supplementary material for: Protein phosphatase PPM1A inhibition attenuates osteoarthritis via regulating TGF-β/Smad2 signaling in chondrocytes
Source: JCI Insight. 2023 Feb 8;8(3):e166688. doi: 10.1172/jci.insight.166688 (PMC9926971; doi:10.1172/jci.insight.166688)
Supplement: Supplemental data [file jciinsight-8-166688-s225.pdf]

Supplemental Material

Protein phosphatase PPM1A inhibition attenuates osteoarthritis *via* regulating TGF-β/Smad2 signaling in chondrocytes

Qinwen Ge[1,2], Zhenyu Shi[1,3], Kai-ao Zou[1,2], Jun Ying[1,3], Jiali Chen[1], Wenhua Yuan[1], Weidong Wang[1,4], Luwei Xiao[1], Xia Lin[5], Di Chen[6], Xinhua Feng[7,*], Ping-er Wang[1,*], Peijian Tong[1,3,*], Hongting Jin[1,8,*]

List of Contents

Supplementary Figure 1. DMM operation induce PPM1A expression in articular cartilage rather than subchodnral bone or synovium.

Supplementary Figure 2. Comparison of skeletal development between WT and PPM1A-KO postnatal mice.

Supplementary Figure 3. PPM1A-KO mice presented normal cartilage homeostasis in adulthood.

Supplementary Figure 4. PPM1A-KO mice exhibited less osteophyte formation but no effect on synovitis in response to DMM surgery.

Supplementary Figure 5. Intra-articular injection of PPM1A inhibitor displayed no therapeutic effect on OA synovitis.

Supplementary Table 1. Primers sequence for RT-qPCR.

Supplementary Table 2. Primers sequence for *PPM1A* genotyping.

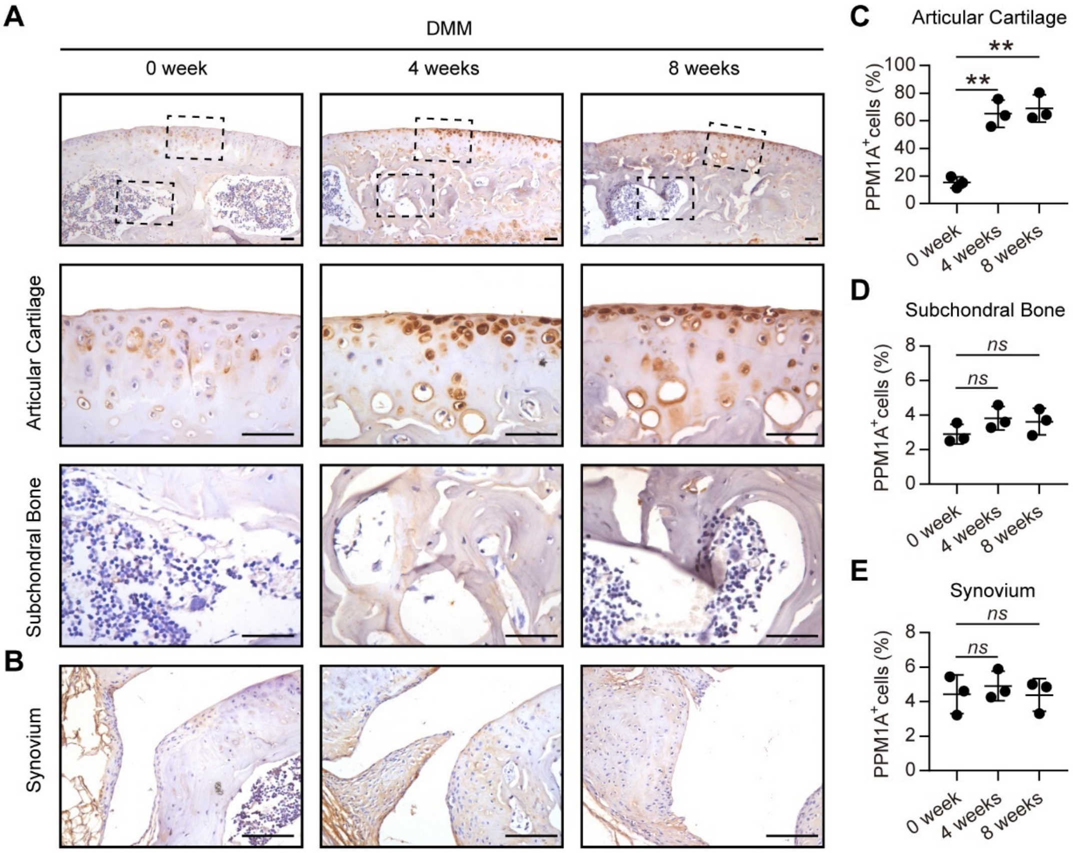

Supplementary Figure 1. DMM operation induce PPM1A expression in articular cartilage rather than subchodnral bone or synovium. (A) Representative immunohistochemical staining for PPM1A in articular cartilage and subchondral bone area at 0 week, 4 weeks, and 8 weeks post DMM initiation. Scale bar = 50 μm. **(B)** Representative immunohistochemical staining for PPM1A in synovium tissue at 0 week, 4 weeks, and 8 weeks after DMM surgery. Scale bar = 100 μm. **(C-E)** Quantitative data of PPM1A positive cells in articular cartilage (C), subchondral bone (D), and syovium (E) at different timepoints. Data were presented as means ± S.D., n = 5 mice at per timepoint, ** = $P < 0.01$ and *ns* = no significance by one-way ANOVA with Dunnett's *t*-test.

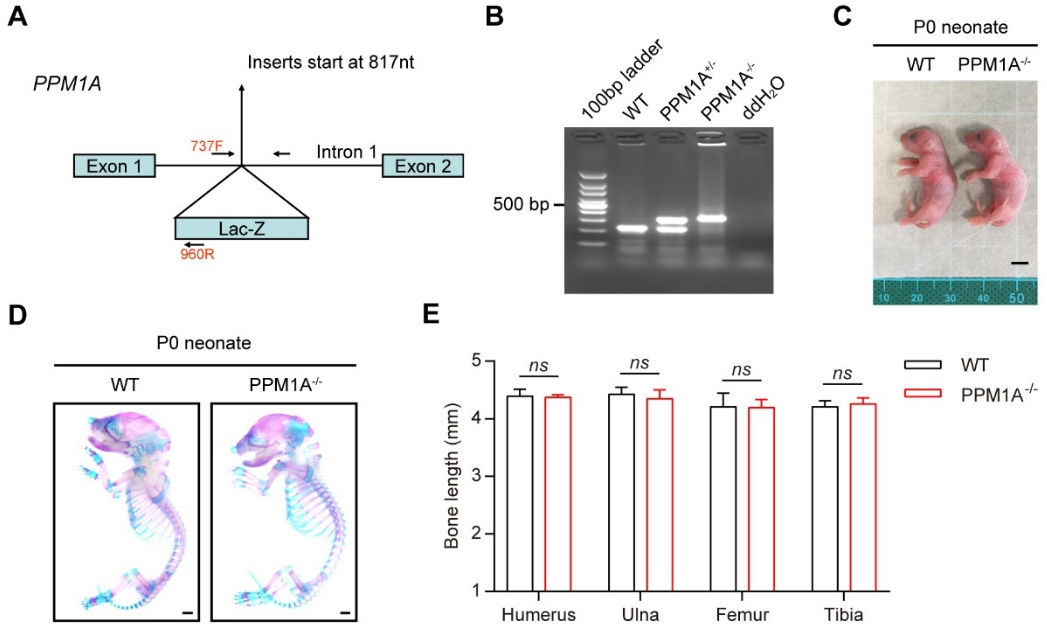

Supplementary Figure 2. Comparison of skeletal development between WT and PPM1A-KO postnatal mice. (A) The schematic diagram of generation of PPM1A-KO mouse. **(B)** Genotyping for WT, PPM1A$^{+/-}$, and PPM1A$^{-/-}$ mice. KO band = 300~400 bp, WT band = 200~300 bp. **(C)** Gross appearance of WT and PPM1A-KO postnatal mice at day 0 (P0). Scale bar = 5 mm. **(D)** Representative images of whole skeleton staining with Alizarin red and Alcian blue of WT and PPM1A$^{-/-}$ mice at P0. Scale bar = 1 mm. **(E)** Quantitative data of long bone length of WT and PPM1A-KO mice at P0. All the data were presented as means ± S.D., n = 3 pups per group, *ns* = no significance by unpaired parametric Student's *t* test.

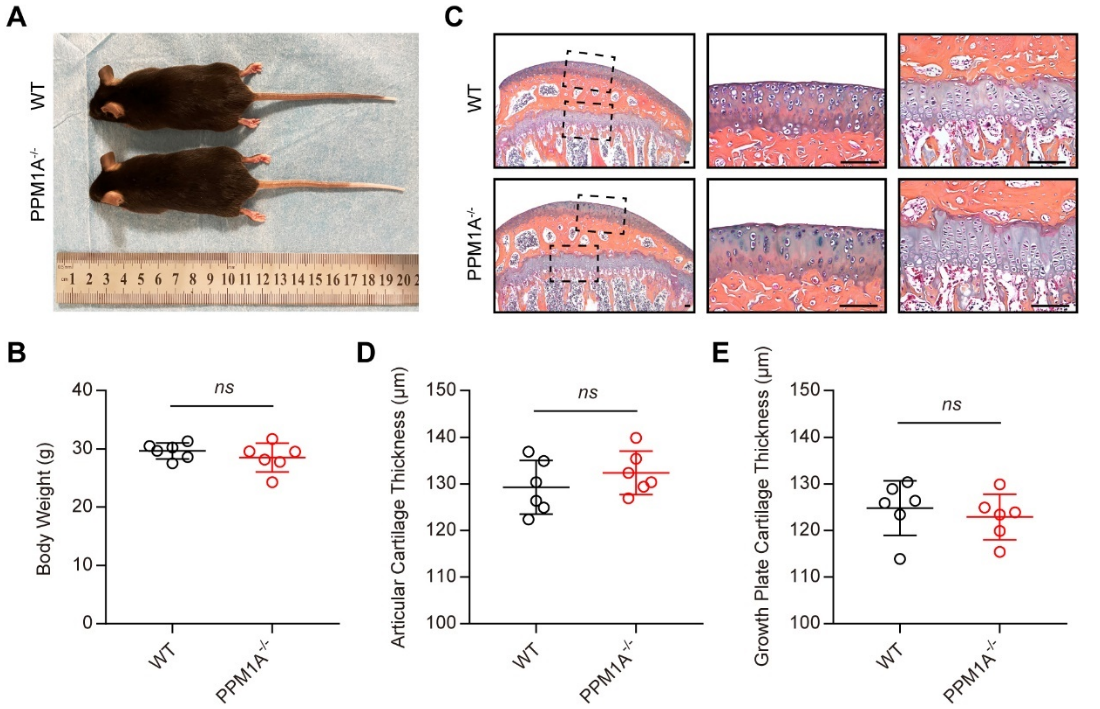

Supplementary Figure 3. PPM1A-KO mice presented normal cartilage homeostasis in adulthood. (A) Gross appearance of male WT and PPM1A-KO mice at 3-month-old. Scale bar = 5 mm. **(B)** Quantification of body weight for male WT and PPM1A-KO mice at 3-month-old. **(C)** ABH/OG staining for both tibial articular cartilage and growth plate from WT and PPM1A$^{-/-}$ mice at 3-month-old. **(D** and **E)** Quantification of articular cartilage thickness (D) and growth plate cartilage thickness (E). Data were presented as means ± S.D., n = 6 mice per group. *ns* = no significance by unpaired parametric Student's *t* test.

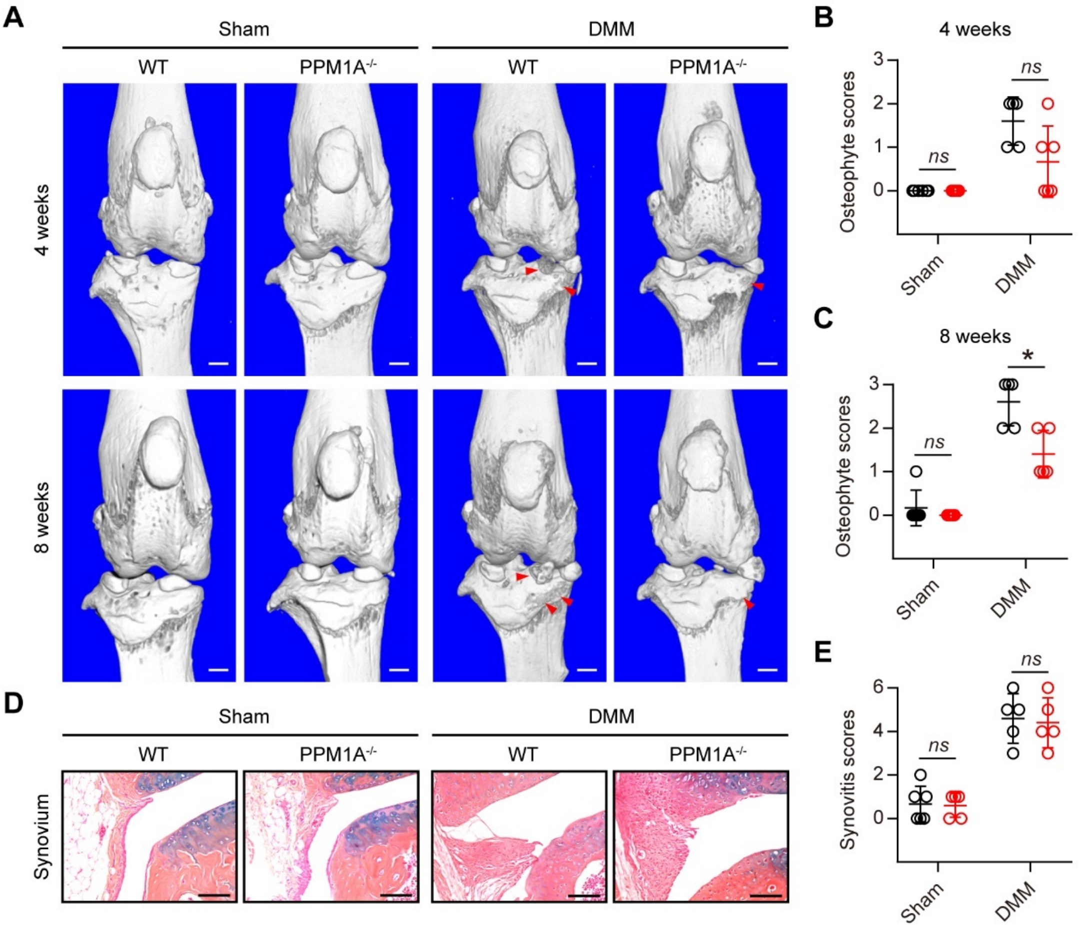

Supplementary Figure 4. PPM1A-KO mice exhibited less osteophyte formation but no effect on synovitis in response to DMM surgery. (A) Representative images of 3D reconstruction for knee joint after sham or DMM surgery at 4 weeks and 8 weeks. Red arrows indicated osteophyte. Scale bar = 1 mm. **(B** and **C)** Quantification of osteophyte scores for gross knee joint at 4 weeks (B) and 8 weeks (C) after operation. **(D)** Representative ABH/OG-stained sections of anterior synovium from WT and PPM1A$^{-/-}$ mice at 8 weeks after sham or DMM surgery. Scale bar = 100 μm. **(E)** Synovitis scores for assessment of the severity of both synovial hyperplasia and synovial inflammation at 8 weeks after surgery. Data were presented as means ± S.D., n ≥ 5 mice per group. * = $P < 0.05$ and *ns* = no significance by two-way ANOVA with Sidak's test.

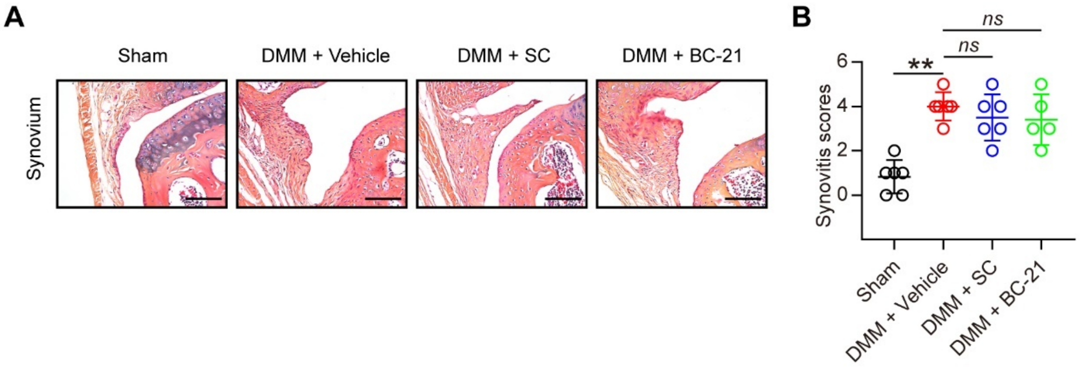

Supplementary Figure 5. Intra-articular injection of PPM1A inhibitor displayed no therapeutic effect on OA synovitis. (A) Representative ABH/OG staining images of anterior synovium from mice treated with vehicel or PPM1A inhibitor at 8 weeks after sham or DMM surgery. Scale bar = 100 μm. **(B)** Synovitis scores for assessment of the severity of both synovial hyperplasia and synovial inflammation at 8 weeks after surgery. Data were presented as means ± S.D., ** = $P < 0.01$ and *ns* = no significance by one-way ANOVA with Dunnett's *t*-test.

Table S1. Primers sequence for RT-qPCR.

Gene	Primer sequences	Origin
PPM1A	Forward: 5’-GGACAAGTACCTGGAGAGCAGA-3’	Mus
	Reverse: 5’-GGGATGTTCTCACTGGCTAACG-3’	Mus
β-actin	Forward: 5’-GGAGATTACTGCCCTGGCTCCTA-3’	Mus
	Reverse: 5’-GACTCATCGTACTCCTGCTTGCTG-3’	Mus

Table S2. Primers sequence for *PPM1A* genotyping.

	Primer sequences
Primer 1	5’-GGCTGAAGCCTGAGAAGGTTC-3’
Primer 2	5’-TCCTCCAGGCAAGTTACAAGC-3’
Primer 3	5’-ATCCTCTGCATGGTCAGGTC-3’
Primer 4	5’-CGTGGCCTGATTCATTCC-3’
